# Socio-Economic Deprivation and Symptom Burden in UK Hospice Patients with Advanced Cancer—Findings from a Longitudinal Study

**DOI:** 10.3390/cancers13112537

**Published:** 2021-05-21

**Authors:** Mari Lloyd-Williams, Christopher Shiels, Christopher Dowrick, David Kissane

**Affiliations:** 1 Academic Palliative and Supportive Care Studies Group, Primary Care and Mental Health, University of Liverpool, Liverpool L69 3BX, UK; cs50@liv.ac.uk (C.S.); Cfd@liverpool.ac.uk (C.D.); 2 Monash Medical Centre, Monash University, Clayton, VIC 3800, Australia; david.kissane@nd.edu.au

**Keywords:** socio-economic deprivation, palliative care, advanced cancer, symptom burden, pain, depression

## Abstract

**Simple Summary:**

We know that socio-economic factors influence delay in presentation and diagnosis of cancer and that patients living in areas of greater socio-economic deprivation are less likely to be referred to palliative care services including hospice. However, very little is known regarding the impact of socio-economic deprivation on symptom burden in advanced cancer patients. Our study found that patients experiencing greater socio-economic deprivation were more likely to report depression and pain and greater global symptom burden than patients from less socio-economically deprived areas. We also found that reporting a lack of information at time of diagnosis was significantly associated with socio-economic deprivation. Although more than one-third of patients recruited into this study were diagnosed with cancer within the preceding 12 months, this was not associated with socio-economic factors and socio-economic factors did not appear to influence survival in our study. The impact of socio-economic factors on symptom burden and information needs should be acknowledged within palliative care settings.

**Abstract:**

Socio-economic deprivation is known to impact on cancer diagnosis, treatment and access to services, but little is known of the impact of socio-economic deprivation on symptom burden in patients with advanced cancer. Patients with advanced cancer attending hospice day services were recruited into a 24 week longitudinal study. An area-based index of social deprivation was collected along with depression and symptom burden at baseline, 8, 16 and 24 weeks. Of the 595 patients included, with an age range of 33–89 years and a mean age of 68 years, 67% were female, and 37% were diagnosed with cancer in the last 12 months. Twenty nine percent lived in one of the most deprived 20% of neighbourhoods. Patients living in the most socio-economically deprived areas were significantly likely to report receiving insufficient information regarding their cancer at diagnosis (*p* = 0.007), greater pain (*p* = 0.02), moderate to severe depression (*p* = 0.04) and higher global symptom burden (*p* = 0.04). This study is the first to report that patients with advanced cancer attending hospice services, living in the most deprived neighbourhoods experience significantly greater symptom burden, notably depression and pain. We recommend using patient outcome measures in order to provide targeted support and thereby reduce the increased symptom burden that socio-economically disadvantaged patients experience at the end of life.

## 1. Introduction

In the UK, there are more than 200 hospices which provide free care to over 225,000 patients living with advanced cancer and other life-limiting conditions. This care includes day care, where patients attend for one day a week for symptom management and support; in-patient ward-based care; and palliative care at home.

Socio-economic factors have been found to influence delay in presentation of cancers. A survey of 2371 patients with different cancers reported that 21% had delayed presentation for 3 months or more and that delay was associated with greater socio-economic deprivation [1]. Socio-economic factors can influence treatment—a Canadian study of 34,022 patients with gastrointestinal advanced cancers found patients living in the most deprived areas were significantly less likely to see a radiation or clinical oncologist after diagnosis and significantly less likely to receive radiotherapy and/or chemotherapy [2].

Palliative care for advanced cancer patients aims to support patients and their families, with particular attention to the physical, psychological, social and spiritual well-being of the patient. Research on inequalities within palliative care has tended to focus on populations and access to care [3,4,5,6] rather than on symptom burden related to socio-economic factors.

Patients from more socio-economically disadvantaged backgrounds are known to be less likely to access home care services providing hospice care at home and less likely to die at home [7,8,9,10]. Analysis of the National UK Survey of Bereaved People [11] revealed that although those living in the first and second most deprived quintiles (based on the index of multiple deprivation (IMD)) had similar access to community based services as those living in areas of less deprivation, those living in areas of greatest deprivation were less likely to die at home. A later survey in 2015 [12] reported that significantly more people in the most deprived areas rated quality of care in the last three months of life as fair or poor (29%) compared with the least deprived group (22%). Research from Australia reported that low socio-economic status was unrelated to inadequate access to palliative care [13] and that patients from the least disadvantaged groups were in fact less likely to be referred to palliative care services. A recent study [14] of 600 patients with stage 4 malignancy in Singapore revealed that patients with greater financial difficulties also reported worse physical, psychological, social and spiritual outcomes.

Analysis of deaths occurring within hospices in England in 1998–2002, 2003–2007, and 2008–2012 compared with those in 1993–1997 [15] found that those living in more socio-economically deprived areas were less likely to die in a hospice in 2008–2012 compared to 1993–1997. An observational study of place of death in Wales also revealed that people living in more socio-economically deprived areas were more likely to die in a hospital [16].

To date, the studies exploring inequalities in palliative care have focused on access to palliative care, and in particular access to hospice provision at the end of life, with studies predominantly using retrospective data [17]. Little is known as to how inequalities impact on symptom burden in advanced cancer within a palliative care population. Additionally, symptom data, both physical and psychological, are limited within existing data sets [18].

We report a large longitudinal study in order to explore how social inequalities impact on symptom burden in patients with advanced cancer attending UK hospice day services.

## 2. Methods

### 2.1. Recruitment

In this paper, we report further data analysis on social inequalities and symptom burden from a large prospective longitudinal study of patients with advanced cancer accessing hospice day services within 20 hospices in the North West of England [19,20], which has areas of significant socio-economic deprivation. The inclusion criteria were consecutive patients with locally advanced or metastatic disease over the age of 18 years and estimated by hospice day care staff to have a prognosis of at least 6 months. Exclusion criteria included any non-cancer diagnosis, insufficient English and cognitive impairment. Eligible patients were informed and provided with written details of what this study entailed by the senior nurse and were invited to contact the researcher if interested in participating. As a research team, we estimated that we would need to recruit 600 eligible patients into this 6 month longitudinal study as attrition was estimated to be 50% and to allow 300 patients to complete at each time point. Baseline data collection interviews at recruitment were mostly conducted in the hospice or occasionally at the patient’s home. Patients usually completed the follow-up questionnaires (PHQ-9 and the co-devised patient-reported outcome scale) by post with a small number opting for researcher contact, which was usually by telephone. Data collection was paper based.

Full ethical approval was obtained (Research Ethics committee 07Q150524).

### 2.2. Information Collected in the Baseline Interview

The baseline interview was carried out by a researcher with patients who consented to be involved with this study and remained with patients whilst they completed the questionnaires offering assistance if required. The baseline questionnaire recorded information regarding each patient’s age, gender, ethnicity, post code of home residence, marital status, primary cancer site, date of cancer diagnosis and the following question was asked “Did you receive sufficient information regarding your cancer illness at the time of diagnosis?”. A baseline assessment of performance status using the response criteria of the Eastern Cooperative Oncology Group (ECOG) [21] was completed. As follow up for this large longitudinal study was mainly remote (postal/telephone), ECOG performance was collected once at baseline.

### 2.3. Postcode and the Index of Multiple Deprivation Score

Each individual postcode was allocated to a census geographical unit, the lower-level super output area (LSOA). Each LSOA has a population of approximately 3000 people and approximately 1200 households. A neighbourhood deprivation score of the LSOA containing the postcode of each patient was generated. LSOA deprivation scores were taken from Indices of Multiple Deprivation for England, which are based on 7 indices, namely, income deprivation, employment deprivation, education, skills and training deprivation, health deprivation and disability, crime, barriers to housing and services, living environment deprivation [22].

The deprivation score was transformed into a national deprivation rank. In England, a rank of one represented the most deprived LSOA in the country, a rank of 32,482 indicated the least deprived LSOA. The national deprivation ranks were then transformed into national quintiles of deprivation, from which it was possible to determine whether a patient was living in the first or second of the 20 percent most deprived LSOAs in their respective country (England or Wales), or the least deprived quintiles.

### 2.4. Psychological and Physical Health Outcomes

Depression was assessed by the Patient Health Questionnaire (PHQ-9), which has validated thresholds for mild (score 5–9), moderate (score 10–14), moderately severe (score 15–19) and severe (score 20–27) depression [23], and has been validated in cancer populations [24]. For the purposes of this study, a cancer patient was deemed to be a depression “case” when scoring 10 or more (i.e., moderate or severe depression) on PHQ-9.

Each patient was invited to indicate the presence by recording Yes/No of any of six individual physical symptoms (pain, breathlessness, nausea, fatigue, constipation, and insomnia) in the previous seven days on a patient-reported outcome scale co-devised with patients for this study and to score their global health rating scale, allowing a combined self-report of subjective severity of all symptoms (1 = least severe, 10 = most severe).

Patients were requested to complete a PHQ-9 and the physical health outcome measures at baseline, 8 weeks, 16 weeks and 24 weeks.

### 2.5. Statistical Analysis

We analysed all the data available and we did not carry out imputation for missing data. At the univariate level of analysis, the chi-square test was used to test the statistical significance of association between baseline patient categorical variables, psychological and physical health outcomes and membership of deprivation categories. The global health rating score was treated as continuous, and a one-way ANOVA test used to compare mean scores across deprivation groups.

Logistic regression models were developed in order to estimate the independent effect of patients living in different areas of deprivation on the risk of being a PHQ-9 depression case or reporting a physical symptom at any time in this study (baseline or one of the follow-up points). In order to take account of differences in the number of times outcome measures were completed by patients, a mixed-effects regression model was used, with time of measurement (baseline, 8 weeks, 16 weeks, 24 weeks) included as an intercept. Unadjusted odds ratios, and estimates adjusted for age, sex and cancer type, are reported for each deprivation quintile. The 95% CIs and associated *p*-values are also reported.

A mixed-effects linear regression model was run in order to estimate the effect of living in different deprivation areas on ratings on the self-rated symptom severity scale. Unadjusted and adjusted β coefficients and *p*-values are reported.

A conventional criterion of statistical significance (*p* < 0.05) was applied in all univariate and multivariate analyses.

Data were analysed using SPSS for Windows 22 (Stanford, CA, USA) and STATA IC10 (California, CA, USA).

## 3. Results

### 3.1. Baseline Characteristics of Patients in This Study

Nine hundred and twenty-one patients who met eligibility criteria were given information regarding this study; 694 (75.4%) patients requested the researcher to contact them and 629 (68.3%) patients agreed to participate. Of these 629 patients, an area social deprivation score could not be assigned to 34 patients due to administrative anomalies in postcode recording.

Of the 595 patients to whom an area social deprivation score could be assigned, with an age range of 33–89 years and a mean age of 68 years, 67% were female, 34% were aged over 70 years, 97% were ‘White British’ and 54% were currently married or cohabiting. In terms of primary cancer diagnosis, 35% had breast cancer, 18% gastrointestinal and 11% lung cancer. Over one-third (37%) of patients had been diagnosed with cancer in the 12 months prior to recruitment. One-quarter of patients scored 3 or 4 on the ECOG scale.

**Figure 1:**
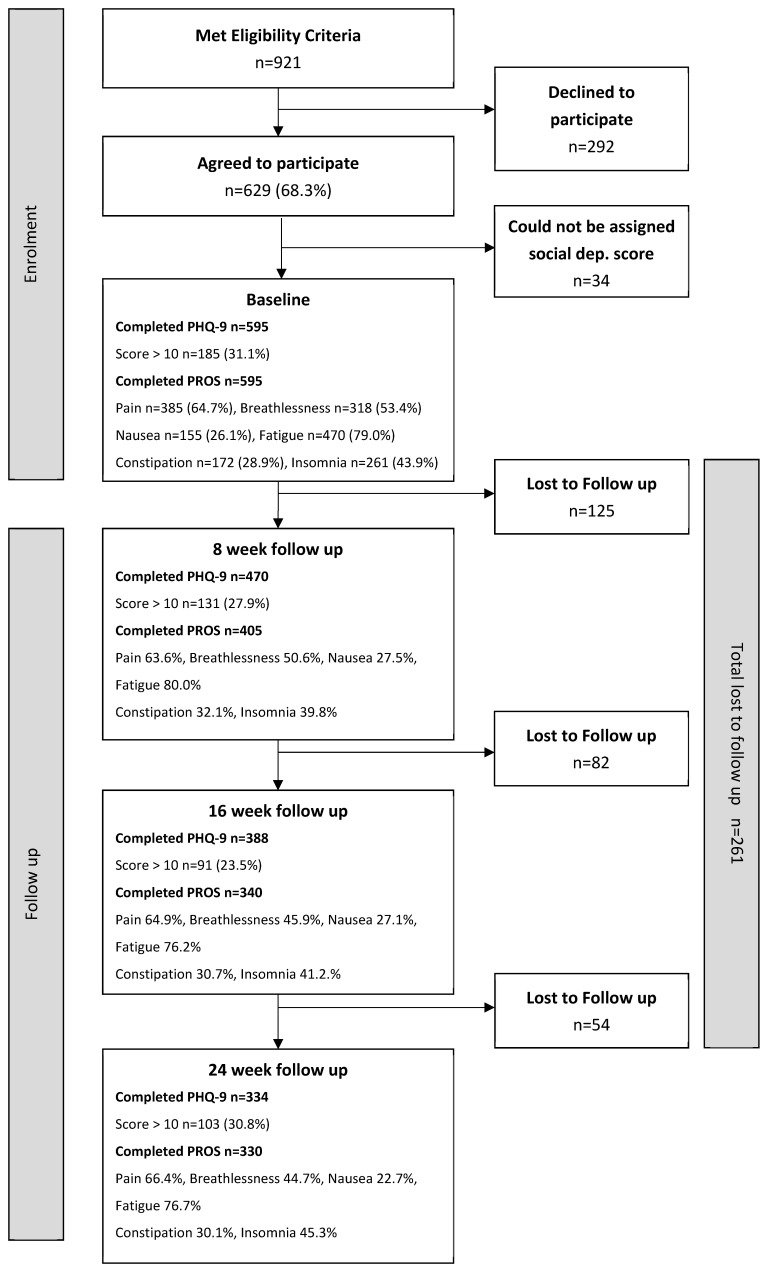
Flowchart.

Twenty nine percent had a home address in one of the most deprived 20% of neighbourhoods in their respective country (England or Wales) and 16% lived in one of the 20% least deprived LSOAs in their country (Figure 1).

### 3.2. Patients Reporting Being a Depression Case

At baseline, 31.1% (185/595) of the cancer patients scored 10 or higher on the PHQ-9, and were classed as depression ‘cases’. At 8 week follow up, 470 patients completed a second PHQ-9 and 27.9% (*n* = 131) scored as depression cases. At 16 weeks and 24 weeks, depression case rates were 23.5% (91/388) and 30.8% (103/334), respectively.

### 3.3. Patients Reporting Physical Health Symptoms

When requested at baseline to report whether experiencing symptoms in the previous 7 days, three hundred and eighty five (64.7%) reported ‘pain’, three hundred and eighteen (53.4%) reported ‘breathlessness’, one hundred and fifty five (26.1%) ‘nausea’, four hundred and seventy (79.0%) ‘fatigue’, one hundred and seventy two (28.9%) ‘constipation’ and two hundred and sixty one (43.9%) ‘insomnia’.

Four hundred and five patients completed patient-reported outcomes at 8 weeks, 340 at 16 weeks and 330 at 24 weeks follow up. At the three follow-up points, symptom burden was relatively similar. The proportions of patients reporting ‘pain’ were 63.6%, 64.9% and 66.4%; ‘breathlessness’ 50.6%, 45.9% and 44.7%; ‘nausea’ 27.5%, 27.1% and 22.7%; ‘fatigue’ 80.0%, 76.2% and 76.7%; ‘constipation’ 32.1%, 30.7% and 30.1%; and ‘insomnia’ 39.8%, 41.2% and 45.3%

Patients were asked to rate global symptom burden in the past 7 days on a 1–10 rating scale. Mean scores at baseline, 8 weeks, 16 weeks and 24 weeks were 4.8, 4.4, 4.5 and 4.6, respectively.

### 3.4. Socio-Economic Deprivation, Patient Characteristics and Health Outcomes

A number of patient characteristics recorded at baseline were associated with the levels of socio-economic deprivation of area of residence (Table 1). Married or cohabiting patients were more likely to live in less deprived areas—39% of married/cohabiting, compared to 27% in other marital status categories, lived in LSOAs in the less deprived quintiles, *p* = 0.01.

There was a significant association between the type of cancer and the residence of the patient (*p* = 0.02). More than half of patients with diagnosis of breast, gastrointestinal, head and neck, and lung cancer resided in the more deprived Q1/2 areas. A higher proportion of patients with a recent cancer diagnosis, within the previous 12 months, were from the most deprived neighbourhoods (52%, compared to 46% of those with a less recent diagnosis), but this was not statistically significant (*p* = 0.20).

Patients scoring 3 or 4 on the baseline ECOG indicating poor performance status were from the most deprived quintiles of LSOAs (54%, compared to 45% of patients scoring 0–2, *p* = 0.04) (Table 2). More than half (54%) of patients scoring 10 or higher on the PHQ-9 at baseline lived in a LSOA within the Q1/2 category (*p* = 0.04). Reporting pain and constipation on the patient-reported outcome scale were significantly associated with area deprivation, with more than 50% of patients reporting pain at baseline were from the most deprived areas (*p* = 0.04). A significantly higher proportion of patients reporting ‘constipation’ were residing in the Q1/2 LSOAs (54% vs. 45% of those not reporting this symptom, *p* = 0.01). Patients living in the most deprived Q1/2 areas had a significantly higher mean rating on the global symptom scale at baseline (5.0, compared to a mean of 4.5 for patients living in Q4/5 neighbourhoods, *p* = 0.04). Patients reporting that they had received sufficient information at diagnosis were more likely to come from the less deprived Q4/5 neighbourhoods *p* = 0.007).

Table 3 reports the results of regression analyses estimating the effects of each of the five deprivation quintiles of patient residential areas on risk of scoring as a depression case or reporting an individual physical symptom at any time in the 24 week measurement period.

Compared to patients living in the least deprived 20% of LSOAs in the country, patients living in the most deprived quintile, Q1, had a significantly increased risk of being a depression case (adjusted OR = 1.75 95% CI 1.04–2.96, *p* = 0.03) and reporting pain (adjusted OR = 1.75 95% CI 1.04–2.96, *p* = 0.02). However, patients in the most deprived quintile areas had a significantly lower likelihood of reporting fatigue compared to those in Q5 (adjusted OR = 0.34 95% CI 0.12–0.96, *p* = 0.04).

Table 4 reports association between residence in each quintile of LSOAs and score on the global rating scale at any time in the study period. Compared to patients living in the least deprived quintile, those residing in quintiles 1, 2 and 3 of the most deprived neighbourhoods experienced significantly greater global symptom burden when compared to those living the least deprived quintile and this was most apparent for those living in the most deprived area quintile 1 (adjusted β = 0.62 95% CI 0.25–0.98, *p* = 0.001).

### 3.5. Socio-Economic Deprivation and Survival

Two hundred and eighteen (36.6%) of the 595 patients included in this analysis died during the study observation period. The mortality rates across the five deprivation groups did not vary significantly (Q1 = 35%, Q2 = 35%, Q3 = 42%, Q4 = 34%, Q5 = 39%, *p* = 0.64). Deaths of patients living in the least deprived quintile tended to be sooner after study entry, with a median survival of 98 days in Q5 with respective median survival times for Q1 to Q4 of 111, 198, 152 and 154 days (*p* = 0.46).

## 4. Discussion

This large longitudinal study of patients with advanced cancer attending UK hospice day care services is unique as it allows exploration of patient outcomes linked to socio-economic deprivation. Nearly one-third of patients in this study lived in the most deprived neighbourhoods in England and Wales and more than half of the patients in this study with breast, lung, gastrointestinal and head and neck cancers resided in the more deprived Q1/2 areas. Moderate to severe depression showed a significant association with socio-economic disadvantage in this study. Within the general population, depression is known to be linked with socio-economic deprivation [25,26]. However, in a previous study of 92 advanced cancer patients receiving palliative care, socio-economic factors had no influence on depression or anxiety when comparing patients receiving palliative care at home and within in-patient hospice settings [27].

Hospice day care settings provide assessment and palliation of physical and psychological symptoms in patients with advanced cancer. During the 24 week study, patients who lived in socio-economically disadvantaged areas were significantly more likely to report pain and constipation than those living in less disadvantaged areas. An association between chronic pain and socio-economic disadvantage [28] and similar association with pain in patients with prostate cancer [29] have been reported, although this latter study focused on early- rather than late-stage disease. Wang et al. [30], in a study of 274 patients in the USA with cancer related pain, of whom 21% had recurrent disease, reported that socio-economic deprivation and treatment-resistant depression were predictive of poor improvement in cancer pain.

A significantly higher global symptom burden score was reported in patients living in more socio-economically deprived areas. A study which collected ESAS data on 120,745 patients newly diagnosed with cancer in Ontario [31] reported that patients in the low-income quintiles had significantly higher odds of moderate-to-severe scores across all symptoms compared with patients in the highest-income quintile. However, this study excluded any patients who had died within 365 days of diagnosis. A cross sectional study of over 1.2 million adults revealed that physical and mental co-morbidities increase with age and are exacerbated by socio-economic deprivation [32]. Information regarding co-morbidities was not routinely collected and such co-morbidities could have contributed to increased global symptom burden in this study. Our findings echo those of a study which found significant effects between economic status of the household for all end of life suffering outcomes in a study in five Asian countries [33].

Patients reporting that they had received sufficient information at diagnosis were significantly more likely to be from less deprived Q4/5 neighbourhoods (*p* = 0.007). Health literacy is known to be lower in patients with greater socio-economic deprivation and can lead to late presentation of cancer, a lack of understanding of information conveyed and reduced adherence to treatment and medication [34]. It has been suggested that strategies to improve health literacy should be implemented to reduce health inequalities. However, there is a debate as to which strategies should be implemented, with further research being required [35]. It is well documented that patient satisfaction with information is related to socio-economic factors. Communication is more directive with less patient involvement arising from clinician’s perceptions that patients from more socio-economically disadvantaged areas do not wish to receive detailed information [36]. Additionally, consultations for patients from more deprived areas are shorter [37]. It would be valuable in further studies to carry out qualitative interviews to allow further exploration of this finding.

There is evidence [38] that patients from more socio-economically disadvantaged areas receive later diagnoses, which impacts on outcomes and survival. In our study, 37% of all patients recruited had received a cancer diagnosis in the preceding 12 months. However, patients from more socio-economically deprived areas did not appear to have worse survival than patients from less socio-economically disadvantaged areas.

## 5. Strengths and Limitations

To our knowledge, this is the first study to report symptom burden longitudinally in a palliative care population linked with socio-economic deprivation and presents new findings which are important for clinicians and policy makers. This was a large multicentre longitudinal study over a 24 week period, which therefore adds greater confidence to our findings. A limitation in this study is that information regarding co-morbidities was not routinely collected, and therefore the impact of co-morbidities could not be determined. We recruited into this study from UK hospices in the North West of England, where 88.4% of the population would describe themselves as White British. We recruited consecutive patients into this study. However, it is known that people from ethnic minority backgrounds access hospice care disproportionately less than White populations and we believe that our study population was typical of hospice populations in the North West of England and indeed of participants in hospice research studies. In future research, we would aim to increase the number of participants from minority groups. It is known that patients from more socio-economically deprived areas may not access hospice services. Our results probably underestimate the true excess symptom burden due to socio-economic deprivation as patients in this study were accessing hospice services and additionally it is possible that a higher proportion of patients from socio-economically deprived areas who were approached may have chosen not to participate in this study.

## 6. Conclusions

This longitudinal study has allowed socio-economic disadvantage within palliative care to be explored from a large longitudinal data set of patient symptom outcomes. We conclude that socio-economic disadvantage is an important factor within palliative care and can influence both physical and psychological symptom burden. Palliative care addresses the holistic needs of patients including social factors. We acknowledge that there is little that can be done to reverse socio-economic disadvantage in late-stage illness. However, by identifying patients and using patient outcome measures, much can be done to provide additional support, with the aim of reducing the increased inequities and magnified symptom burden that socio-economically disadvantaged patients experience at the end of life.

## Figures and Tables

**Table 1:** Area of residence, socio-demographic and diagnostic characteristics of cancer patients.

Characteristics	Neighbourhood (LSOA) of Residence			
	Higher Deprivation	Intermediate Deprivation	Lower Deprivation	*p*-Value
−	Q1/Q2	Q3	Q4/Q5	
−	% (*n*/*N*)	% (*n*/*N*)	% (*n*/*N*)	*p*
**Gender**				
Male	47.9 (93/194)	16.0 (31/194)	36.1 (70/194)	0.38
Female	47.9 (192/401)	20.2 (81/401)	31.9 (128/401)	
**Age group**				
Aged under 60	51.2 (83/162)	18.5 (30/162)	30.2 (49/162)	0.35
Aged 60–70	42.2 (86/204)	21.1 (43/204)	36.8 (75/204)	
Aged over 70	50.9 (116/228)	17.1 (39/228)	32.0 (73/228)	
**Marital status**				
Married/cohabiting	40.8 (130/319)	20.1 (64/319)	39.2 (125/319)	0.01
Divorced/widowed/single (living alone)	55.4 (113/204)	18.6 (38/204)	26.0 (53/204)	
Divorced/widowed/single (not living alone)	56.4 (31/55)	16.4 (9/55)	27.3 (15/55)	
**Type of cancer**				
Breast	44.9 (92/205)	17.6 (36/205)	37.6 (77/205)	0.02
Gastrointestinal	57.1 (60/105)	17.1 (18/105)	25.7 (27/105)	
Lung	57.4 (39/68)	23.5 (16/68)	19.1 (13/68)	
Head and neck	62.5 (20/32)	15.6 (5/32)	21.9 (7/32)	
Male specific	40.7 (22/54)	13.0 (7/54)	46.3 (25/54)	
Female specific	42.0 (21/50)	26.0 (13/50)	32.0 (16/50)	
Other	38.3 (31/81)	21.0 (17/81)	40.7 (33/81)	
**Time since cancer diagnosis**				
In previous 12 months	51.6 (113/219)	19.6 (43/219)	28.8 (63/219)	0.20
More than 12 months	45.7 (172/376)	18.4 (69/376)	35.9 (135/376)	
**Given sufficient information at diagnosis**				
No	50.0 (93/186)	22.6 (42/186)	27.4 (51/186)	0.007
Yes	47.2 (185/392)	16.8 (66/392)	36.0 (141/392)	

**Table 2:** Area of residence, activity status, reported physical symptoms and depression at baseline.

Neighbourhood (LSOA) of Residence				
Characteristics	Higher Deprivation	Intermediate Deprivation	Lower Deprivation	*p*-Value
−	Q1/Q2	Q3	Q4/Q5	
−	% (*n*/*N*)	% (*n*/*N*)	% (*n*/*N*)	*p*
**ECOG score**				
0/1	45.1 (128/284)	19.7 (56/284)	35.2 (100/284)	0.04
2	47.5 (76/160)	23.8 (38/160)	28.8 (46/160)	
3–4	53.6 (81/151)	11.9 (18/151)	34.4 (52/151)	
**Depression**				
PHQ score < 10	44.6 (183/410)	19.3 (79/410)	36.1 (148/410)	0.04
PHQ score ≥ 10	55.1 (102/185)	17.8 (33/185)	27.0 (50/185)	
**Reported physical symptom**				
Pain				
No	43.3 (91/210)	16.2 (34/210)	40.5 (85/210)	0.02
Yes	50.4 (194/385)	20.3 (78/385)	29.4 (113/385)	
Breathlessness				
No	46.2 (128/277)	18.1 (50/277)	35.7 (99/277)	0.49
Yes	49.4 (157/318)	19.5 (62/318)	31.1 (99/318)	
Nausea				
No	50.0 (220/440)	17.7 (78/440)	32.3 (142/440)	0.21
Yes	41.9 (65/155)	21.9 (34/155)	36.1 (56/155)	
Fatigue				
No	48.8 (61/125)	17.6 (22/125)	33.6 (42/125)	0.92
Yes	47.7 (224/470)	19.1 (90/470)	33.2 (156/470)	
Constipation				
No	45.4 (192/243)	17.7 (75/423)	36.9 (156/423)	0.01
Yes	54.1 (93/172)	21.5 (37/172)	24.4 (42/172)	
Insomnia				
No	48.8 (163/334)	20.1 (67/334)	31.1 (104/334)	0.41
Yes	46.7 (122/261)	17.2 (45/261)	36.0 (94/261)	
**No of reported physical symptoms**				
0–3	46.3 (174/376)	17.6 (66/376)	36.2 (136/376)	0.14
4–6	50.7 (111/219)	21.0 (46/219)	28.3 (62/219)	
**Global symptom severity rating (mean) (95% CI)**	5.0 (4.7–5.3)	4.9 (4.5–5.3)	4.5 (4.3–4.7)	0.04

**Table 3:** Risk of being a depression case or reporting individual physical symptom in 24 week study period: patients living in the most deprived quintile of LSOAs (Q1) compared to those in least deprived quintile (Q5).

Characteristics	Unadjusted OR (95% CI)	*p*	Adjusted * OR (95% CI)	*p*
**Depression case (PHQ ≥ 10)** Q1 vs. Q5	1.81 (1.09–3.01)	0.02	1.75 (1.04–2.96)	0.03
**Pain** Q1 vs. Q5	1.99 (1.01–3.93)	0.04	2.18 (1.09–4.39)	0.03
**Breathlessness** Q1 vs. Q5	1.16 (0.68–1.97)	0.58	1.18 (0.68–2.07)	0.54
**Nausea** Q1 vs. Q5	0.75 (0.45–1.25)	0.26	0.66 (0.39–1.12)	0.12
**Fatigue** Q1 vs. Q5	0.35 (0.13–0.94)	0.04	0.34 (0.12–0.96)	0.04
**Constipation** Q1 vs. Q5	1.79 (1.07–2.99)	0.03	1.75 (1.04–2.96	0.02
**Insomnia** Q1 vs. Q5	1.04 (0.62–1.74)	0.89	0.99 (0.58–1.72)	0.99

* Odds ratio adjusted for age, sex and type of cancer.

**Table 4:** Independent association between social deprivation of residential neighbourhood and score on self-rated global symptom burden scale in 24 week study period.

Residence in LSOA	Unadjusted β (95% CI)	*p*	Adjusted β (95% CI)	*p*
**Social deprivation quintile**	−	−	−	−
**Q5 (least deprived)**	1.00	−	1.00	−
**Q4**	0.14 (−0.76, 0.52)	0.46	0.12 (−0.74, 0.50)	0.54
**Q3**	0.49 (0.11, 0.87)	0.01	0.48 (0.09, 0.86)	0.01
**Q2**	0.47 (0.10, 0.85)	0.01	0.45 (0.07, 0.84)	0.02
**Q1 (most deprived)**	0.62 (0.27, 0.97)	0.001	0.62 (0.25, 0.98)	0.001

## Data Availability

All Data supporting this study has been archived at the University of Liverpool according to research ethics requirements.
